# Wnt3a promotes radioresistance via autophagy in squamous cell carcinoma of the head and neck

**DOI:** 10.1111/jcmm.14394

**Published:** 2019-05-21

**Authors:** Qiancheng Jing, Guo Li, Xiyu Chen, Chao Liu, Shanhong Lu, Hua Zheng, Huiling Ma, Yuexiang Qin, Diekuo Zhang, Shuiting Zhang, Shuling Ren, Donghai Huang, Pingqing Tan, Jie Chen, Yuanzheng Qiu, Yong Liu

**Affiliations:** ^1^ Department of Otolaryngology Head and Neck Surgery Xiangya Hospital, Central South University Changsha People’s Republic of China; ^2^ Otolaryngology Major Disease Research Key Laboratory of Hunan Province Changsha Hunan People’s Republic of China; ^3^ Department of Otolaryngology Head and Neck Surgery Changsha Central Hospital, University Of South China Changsha Hunan People’s Republic of China; ^4^ Health Management Center Third Xiangya Hospital, Central South University Changsha People’s Republic of China; ^5^ Department of Head and Neck Surgery, Hunan Cancer Hospital, The Affiliated Tumor Hospital of Xiangya Medical School Central South University Changsha Hunan People’s Republic of China

**Keywords:** autophagy, prognosis, radioresistance, squamous cell carcinoma of the head and neck, Wnt3a

## Abstract

The canonical Wnt/β‐catenin signalling pathway and autophagy play critical roles in cancer progression. However, the role of Wnt‐mediated autophagy in cancer radioresistance remains unclear. In this study, we found that irradiation activated the Wnt/β‐catenin and autophagic signalling pathways in squamous cell carcinoma of the head and neck (SCCHN). Wnt3a is a classical ligand that activated the Wnt/β‐catenin signalling pathway, induced autophagy and decreased the sensitivity of SCCHN to irradiation both in vitro and in vivo. Further mechanistic analysis revealed that Wnt3a promoted SCCHN radioresistance via protective autophagy. Finally, expression of the Wnt3a protein was elevated in both SCCHN tissues and patients' serum. Patients showing high expression of Wnt3a displayed a worse prognosis. Taken together, our study indicates that both the canonical Wnt and autophagic signalling pathways are valuable targets for sensitizing SCCHN to irradiation.

## 1 INTRODUCTION

Squamous cell carcinoma of the head and neck (SCCHN) accounts for more than 90% of head and neck cancers and is the sixth most common type of cancer worldwide. Radiotherapy is one of the primary therapeutic choices for patients with SCCHN, particularly for those in late and/or advanced stages. However, intrinsic and acquired radioresistance is a major limitation and contributes to treatment failure, disease progression and relapse in patients with SCCHN.^1^, ^2^ To overcome these issues, studies of the molecular mechanisms of radioresistance, which is important for the discovery and development of novel radiation sensitizers for cancer radiotherapy, are urgently needed.

Wnt signalling is essential for stem cell regulation during development and tissue homoeostasis.^3^ Mutated components in the Wnt pathway cause multiple growth‐related pathologies and cancer.^4^ In the canonical Wnt/β‐catenin signalling pathway (hereafter referred to as the Wnt signalling pathway), diverse Wnt ligands bind to Frizzled receptors and low‐density lipoprotein‐related protein 5/6 co‐receptors, stabilizing β‐catenin protein by inhibiting the function of the protein destruction complex constituted by adenomatous polyposis coli, Axin, casein kinase 1 and glycogen synthase kinase 3. Stabilized β‐catenin enters the nucleus and replaces T cell factor‐associated co‐repressors Groucho with coactivators such as TCF/LEF, leading to the transcriptional activation of β‐catenin target genes such as the Cyclin D1, c‐Myc and Survivin genes.^3^, ^4^ The activated Wnt signalling pathway up‐regulates multiple genes such as aldehyde dehydrogenase,^5^ DNA ligase 4,^6^ high mobility group box 1 7 and functions critically in cancer malignant behaviours including radioresistance. However, the underlying molecular mechanisms of Wnt signalling‐mediated radioresistance remain unclear.

Autophagy is a dynamic cellular metabolism process that depends on the lysosome to reutilize proteins and damaged organelles.^8^ Autophagy plays a protective role in cells and tissues under stress conditions. Irradiation induces autophagy to sustain the survival of cancer cells, thereby contributing to treatment resistance and recurrence.^9^ Inhibiting the autophagic response using specific inhibitors or by targeting autophagy‐associated genes has shown efficacy for sensitizing cancer cells to radiotherapy.^10^ Multiple clinical trials targeting autophagy as part of a combination therapy strategy in diverse cancers are undergoing and have shown encouraging results, highlighting the importance of clarifying the regulatory mechanisms of autophagy and accelerating the discovery of molecular targets for selective and specific inhibition aimed at autophagy.^11^

However, the role of Wnt‐mediated autophagy in cancer radioresistance is unclear. In this study, we demonstrated that irradiation induced the activation of both the Wnt/β‐catenin and autophagic signalling pathways in SCCHN cells. Wnt3a‐mediated activation of the Wnt/β‐catenin signalling pathway and autophagy decreased the sensitivity of SCCHN cells to irradiation both in vitro and in vivo. Further analysis of the mechanism revealed that Wnt3a promoted SCCHN radioresistance by promoting protective autophagy. Finally, the expression of Wnt3a protein was elevated in both SCCHN tissues and patients' serum. Patients with high expression of Wnt3a displayed a worse prognosis. Taken together, our results indicate that both the canonical Wnt and autophagic signalling pathways are valuable targets that sensitize SCCHN to irradiation.

## 2 MATERIALS AND METHODS

### 2.1 Cell culture

The human SCCHN cell line Tu686 was kindly provided by Dr Zhuo Georgia Chen (Winship Cancer Institute, Emory University School of Medicine, Atlanta, GA, USA).^12^ The nasopharyngeal carcinoma cell line 6‐10B was purchased from the Cell Center of Central South University (Changsha, China). As we previously described, gradually increased doses of irradiation were administered to nasopharyngeal carcinoma 6‐10B cells to screen and establish 6‐10B cells with enhanced radioresistant capacity (abbreviated as 6‐10B‐Rs).^13^, ^14^ Tu686 cells were maintained in DMEM/F12 (1:1). 6‐10B and 6‐10B‐Rs cells were cultured in RPMI1640 medium. All media were supplemented with 10% foetal bovine serum, 100 IU/mL penicillin and 100 μg/mL streptomycin at 37°C in a humidified atmosphere with 5% CO_2_. Cell lines were routinely excluded for mycoplasma contamination and cells in the exponential phase were used in the following experiments.

### 2.2 Wnt3a overexpression or Beclin1 knockdown in SCCHN cells

To overexpress Wnt3a, full‐length human Wnt3a cDNA was amplified by PCR and then cloned into the pLV lentiviral plasmid (Addgene, Watertown, MA, USA). An empty vector was used as a control. For Beclin1 knockdown, four siRNA were used and target sequences were as follows: siRNA 1:5′‐GGA GAT CTT AGA GCA AAT GAA‐3′, siRNA 2:5′‐GGA CAC GAG TTT CAA GAT CCT‐3′, siRNA 3:5′‐CCA ACG TCT TTA ATG CAA CCT‐3′, siRNA 4:5′‐CCA ATA AGA TGG GTC TGA AAT‐3′. After validation, the sequence of siRNA 1 with the highest inhibitory efficiency was chosen to design an shRNA and package the lentivirus. Target shRNA was cloned into the shRNA‐expressing lentiviral vector pLKO.1 (Addgene). The above vectors together with lentivirus packaging vectors were cotransfected into 293T cells using FuGENE^®^ 6 (Promega, Madison, WI, USA). Lentiviral particles were harvested 48 hours after transfection and used for cell transfection. Stable cells were screened with puromycin for 2 weeks and the efficiency of gene modulation was quantified by Western blotting assays.

### 2.3 Irradiation

Irradiation (IR) was delivered at room temperature using a 6‐MeV electron beam generated by a linear accelerator (2100EX, Varian Medical Systems, Palo Alto, CA, USA) at a dose rate of 300 cGy/min. Compensation glue (1.5‐cm thick) was used to cover the cell culture containers. The source‐to‐skin distance was 100 cm.

### 2.4 Immunoblotting assays

Protein from tumour cells, xenograft samples and human SCCHN samples was extracted using RIPA buffer (10 mmol L^−1^ Tris‐Cl (pH 8.0), 1 mmol L^−1^ EDTA, 1% Triton X‐100, 0.1% sodium deoxycholate, 0.1% SDS, 140 mmol L^−1^ NaCl, 1 mmol L^−1^ PMSF) supplemented with inhibitors of protease and phosphatase. Western blotting assays were then performed as we described previously.^14^, ^15^, ^16^ Briefly, protein samples were separated in 8%‐12% SDS‐PAGE and transferred onto a polyvinylidene fluoride membrane (Millipore, Bedford, MA, USA). Blotted membranes were incubated with primary antibodies at 4°C overnight or for 2 hours at room temperature and then secondary antibodies for 1 hours at room temperature. GAPDH was used as a loading control. Protein bands were visualized using enhanced chemiluminescence reagents and images were captured with Image Lab 4.1 (Bio‐Rad, Hercules, CA, USA). Each experiment was performed in duplicate or triplicate. The antibody working concentration, catalogue number, company source and reaction species are listed in Table S1.

### 2.5 Plate clonogenic survival assays

Radioresistance was measured by clonogenic survival assay following exposure to different doses of irradiation. Briefly, 300‐600 cells were seeded into 6‐well plates and exposed to specific doses of irradiation. After irradiation, the cells were cultured for another 12‐14 days and the number of surviving colonies (defined as a colony with > 50 cells) was counted. The survival fraction was calculated as we previously described.^14^, ^17^ Each experiment was performed in duplicate or triplicate.

### 2.6 Immunofluorescence microscopy analysis

SCCHN cells from different groups were grown on glass coverslips (12 mm) in 24‐well plates. To compare irradiation‐induced foci of γH2AX, the cells were subjected to 4‐Gy irradiation and compared to non‐irradiated control cells 6 hours post‐irradiation. Slide cells were fixed with 4% paraformaldehyde for 15 min at room temperature and then permeabilized with 0.2% Triton X‐100 in PBS for 10 min at room temperature. After treatment with blocking solution, the cell slides were incubated with a rabbit monoclonal antibody against γH2AX (Santa Cruz Biotechnology, Dallas, TX, USA) overnight at 4°C. After washing with staining buffer (PBS, bovine serum albumin (1%), glycine (0.15%), Triton X‐100 (0.1%)), the cells were incubated with secondary antibody (Dylight594 IgG antibody, Genetex, Irvine, CA, USA). Nuclei were counterstained with DAPI (Sigma, St. Louis, MO, USA) for 5 min at room temperature and washed with staining buffer. The coverslips were mounted onto glass slides with anti‐fade solution. Final images were captured with a Leica wide‐field microscope (Wetzlar, Germany). The number of γH2AX foci was determined by counting at least 1000 cells per condition in at least three randomly selected fields and quantified using ImageJ software (NIH, Bethesda, MD, USA).^18^Each experiment was performed in duplicate or triplicate.

### 2.7 Treatment with small molecular compounds

The small synthetic molecules 3‐methyladenine (3MA) (TargetMol Corp., Boston, MA, USA) and recombinant human Wnt3a protein (rhWnt3a) (Abnova, CA, USA) were used to inhibit autophagy or activate the Wnt/β‐catenin signalling pathway. 3MA blocks autophagy via inhibiting the PI3K class III. SCCHN Tu686 and 6‐10B cells from different groups were treated with 800 µM 3MA and 20 ng/mL rhWnt3a for the indicated times and then subjected to a clonogenic survival assay, Western blotting assay and immunofluorescence staining.

### 2.8 Xenograft tumour model

Athymic male nude mice were purchased from Hunan SJA Laboratory Animal Co., Ltd. (Changsha, Hunan, China). Mice were housed in a specific pathogen‐free laboratory. Animal procedures were reviewed and approved by the Animal Ethics Committee of Central South University (Changsha, Hunan, China). Mice at the age of 5‐7 weeks were subcutaneously injected into the hind flank with SCCHN cells (indicated SCCHN cells transfected with Wnt3a shRNA and control cells) suspended in 200 µL cold PBS. The animals were then subjected to irradiation or autophagy inhibitor 3MA treatment (30 mg/kg) alone or together when the tumours reached a volume of 100 mm^3^. Mice in the irradiation‐treated group were subjected to a total of 8 Gy irradiation in 4‐Gy doses for 2 consecutive days. Non‐treated mice were sham‐irradiated. Basic conditions and individual mouse weights were monitored throughout the study period. Tumour volume was determined every 3‐4 days with a calliper and calculated using the modified ellipse formula (volume = length × width^2^/2). Two weeks after the first irradiation exposure, all mice were killed. Xenograft tumours in all groups were removed and the final individual weight of each tumour was measured. Each tumour sample was cut into two parts and fixed with formaldehyde or stored in nitrogen. Tumour samples were used for haematoxylin and eosin staining, Western blotting assay and immunostaining.

### 2.9 SCCHN patient information and tissue preparation

One hundred thirty‐eight paraffin‐embedded SCCHN patient samples from the Department of Otolaryngology in Xiangya Hospital and Department of Head and Neck Surgery, Hunan Cancer Hospital and The Affiliated Tumor Hospital of Xiangya Medical School, Central South University were used to analyse the clinical significance of Wnt3a and Beclin1. Samples were collected from January 2002 to October 2004 from patients with no history of previous malignancies or radio‐ or chemotherapy. Recurrence and metastasis were established based on physical examinations, imaging results, operation and post‐operative pathological examination. Informed consent was obtained from all patients before surgery. Pathological tumour‐node‐metastasis stage was determined according to the 7th American Joint Committee on Cancer staging system. Patient characteristics such as sex, age, primary tumour sites, clinical stages and lymph node metastasis are shown in Table 1. Overall survival was calculated from the day of surgery to the date of death. Death from other causes was treated as censored cases. The follow‐up time for the patient cohort was 5 years.

**Table 1:** Correlation of Wnt3a expression with clinicopathological features in 138 patients with SCCHN

Parameters	Low	Middle	High	*χ2* value	*P*‐value^a^
Sex					
Female	1	5	0	2.885	0.236
Male	53	64	15		
Age					
<58	24	37	7	1.067	0.587
≥58	30	32	8		
Primary tumour sites					
Glottic	38	43	11	1.220	0.543
Others	16	26	4		
T classifications					
T1 + T2	28	30	3	4.858	0.088
T3 + T4	26	39	12		
Lymph node metastasis					
N0	44	41	10	6.910	**0.032**
N+	10	28	5		
Histological grades					
G1 + G2	35	49	14	4.638	0.098
G3 + G4	19	20	1		
Clinical stages					
I	5	4	0	18.869	**0.004**
II	23	16	2		
III	23	27	7		
IV	3	22	6		

a *P* < 0.05 was considered to be statistically significant, in which significant *P*‐values were indicated in bold.

Additionally, 108 serum samples from patients with SCCHN and healthy donors were collected from March 2018 to October 2018 to quantify the serum level of Wnt3a. The study was approved by the Research Ethics Committee of Central South University, Changsha, China.

### 2.10 Haematoxylin and eosin staining, immunohistochemistry and quantification

Four‐micrometre thick paraffin‐imbedded tumour sections were initially stained with haematoxylin and eosin to confirm the tumourigenesis of SCCHN cells in vivo. All immunohistochemistry staining was performed as described in our previous studies.^19^, ^20^, ^21^, ^22^ Briefly, human SCCHN and xenograft sections were stained with the indicated primary antibodies, followed by sequential incubation of secondary antibody and diaminobenzidine. Slides were incubated with immunoglobulin G rather than primary antibodies as negative controls.

To evaluate human SCCHN samples, staining intensity was scored as 0 (negative), 1 (weak), 2 (medium) and 3 (strong). The degree of staining was scored as 0 (0%), 1 (1%‐25%), 2 (26%‐50%), 3 (51%‐75%) and 4 (76%‐100%), the sum of staining intensity (0‐3) and degree (0‐4) was used to determine the final staining score (0‐7). Patients with SCCHN were categorized into three distinct groups including low (0‐2), middle (3‐4) and high (5‐7) expression groups based on the final staining scores.^19^, ^20^, ^21^, ^22^

### 2.11 Enzyme‐linked immunosorbent assay (ELISA)

Serum Wnt3a expression levels in 108 samples from patients with SCCHN were quantified according to the manufacturer's instructions of the Human Protein Wnt3a ELISA kit (Cat. CSB‐EL026136HU, Cusabio Technology, Houston, TX, USA).^23^

### 2.12 Statistical analysis

Statistical analyses were performed with SPSS software version 17.0 (SPSS, Inc, Chicago, IL, USA). Quantitative data in this study are expressed as the mean ± SD. Significant differences between groups were determined by analysis of variance. The Chi‐squared test was used for statistical analysis of categorical data. Survival curves were constructed using the Kaplan‐Meier method and evaluated using the log‐rank test. Additionally, the Cox proportional hazard regression model was used to identify factors that were independently associated with overall survival. The Spearman rank correlation coefficient was employed to determine whether there was a correlation between the expression of Wnt3a and Beclin1. A *P*‐value < 0.05 was considered statistically significant in a two‐tailed test.

## 3 RESULTS

### 3.1 Irradiation activates Wnt signalling pathway and induces autophagy in SCCHN

**Figure 1:**
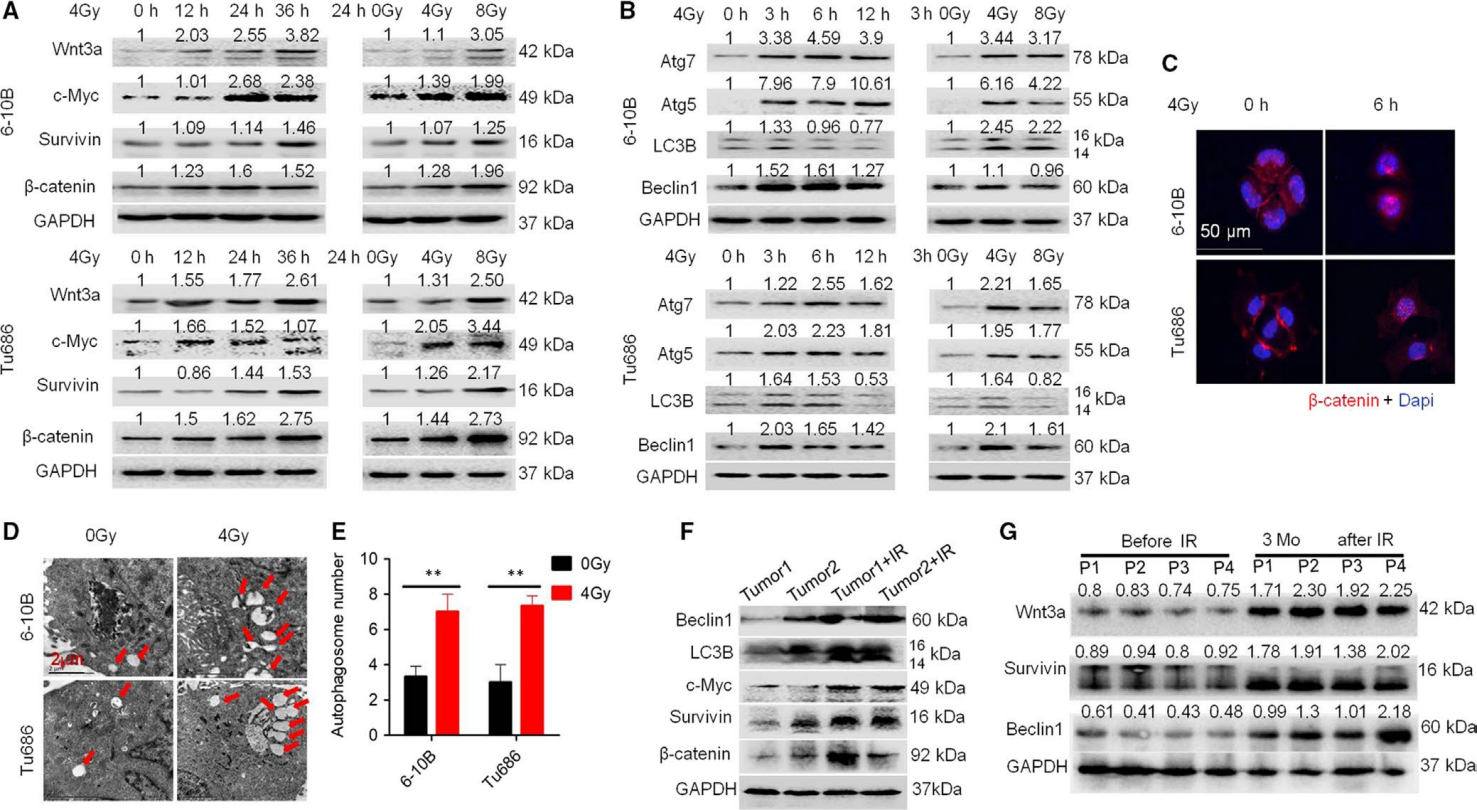
Irradiation activates Wnt signalling pathway and induces autophagy in SCCHN. Western blotting assays were used to evaluate the expression of proteins in the Wnt (A) and autophagy (B) signalling pathways in SCCHN 6‐10B and Tu686 cells exposed to 4 Gy irradiation and examined at different times (0, 12, 24 and 36 hours; Right panel) or subjected to different doses (0, 4 and 8 Gy) of irradiation and examined at 24 hours (Left panel). (C) Immunofluorescence staining was used to confirm the nuclear translocation of β‐catenin in 6‐10B and Tu686 cells at 6 hours post‐irradiation with 4 Gy irradiation. (D) Representative images of autophagosomes (indicated by red arrows) observed by transmission electron microscopy in 6‐10B and Tu686 cells at 3 hours post 4 Gy irradiation exposure. (E) Quantification of autophagosomes in 6‐10B and Tu686 cells. (F) 6‐10B cells were subcutaneously injected into the flanks of nude mice and irradiated with 8 Gy irradiation. Two weeks post‐irradiation, Western blotting assays were conducted to quantify the expression of proteins in the Wnt and autophagy signalling pathways. (G) Expression of Wnt3a, Beclin1 and Survivin in four cases of SCCHN tissues before and after radiotherapy as assayed by Western blotting assay. IR stands for irradiation. **P < 0.01

Our previous RNA‐sequencing data revealed that radioresistant 6‐10B‐Rs cells displayed differentially expressed genes compared to the parent 6‐10B cells.^14^ Kyoto Encyclopedia of Genes and Genomes analysis of these differentially expressed genes indicated that the Wnt signalling pathway was one of the most enriched signalling pathways (Figure S1A). Additionally, continuous irradiation increased the expression of multiple Wnt ligands, in which Wnt3a as a canonical ligand of the Wnt signalling pathway, was significantly up‐regulated (Figure S1B). Consistent with this result, SCCHN Tu686 and 6‐10B cells displayed gradually increased expression of β‐catenin protein and its downstream targets c‐Myc and Survivin following exposure to 4 Gy irradiation at different time points and stimulated by increased doses of irradiation at 24 hours (Figure 1A), which was accompanied by translocation of β‐catenin into the nucleus in both Tu686 and 6‐10B cells at 6 hours after irradiation exposure (Figure 1C). More importantly, 6‐10B cells similarly showed up‐regulated expression of these proteins associated with the Wnt signalling pathway when subcutaneously injected into nude mice and subjected to a total of 8 Gy irradiation in vivo (Figure 1F). Overall, these data demonstrate that irradiation induces activation of the Wnt signalling pathway in SCCHN cells.

We also investigated the alterations in the autophagic cascades. As shown in Figure 1B, autophagic signalling proteins including Beclin1, Atg5, Atg7 and LC3B were up‐regulated in a time‐ and dose‐dependent manner by irradiation stimulation. Transmission electron microscopy (TEM) analysis revealed that irradiation exposure increased the number of autophagosomes in Tu686 and 6‐10B cells (Figure 1D and 1). Xenograft tumours of 6‐10B also showed elevated expression of Beclin1 and LC3B following irradiation stimulation (Figure 1F). Finally, radioresistant 6‐10B‐Rs cells established by exposure to gradually increased doses of irradiation for 6 months also displayed activation of the Wnt and autophagic signalling pathways (Figure S1C‐E). These results indicate that irradiation activates the Wnt signalling pathway and induces autophagy in SCCHN cells.

### 3.2 Wnt3a enhances radioresistance and autophagy in SCCHN

**Figure 2:**
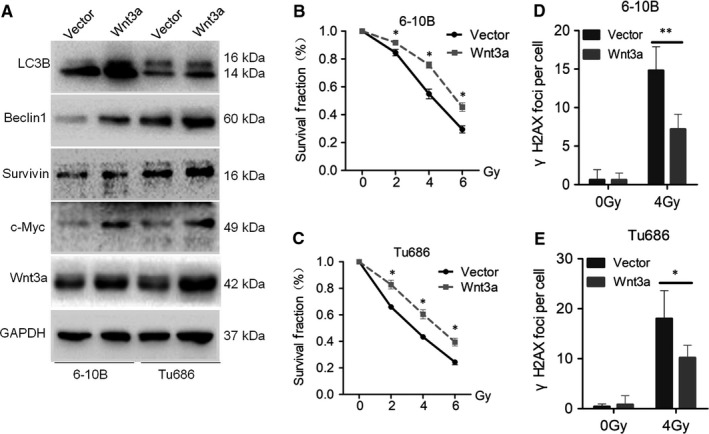
Wnt3a enhances radioresistance and autophagy in SCCHN. SCCHN 6‐10B and Tu686 cells were infected with lentivirus‐mediated Wnt3a or control cDNA and then subjected to puromycin screening for 2 weeks. (A) Western blotting was used to examine the expression of proteins associated with the Wnt and autophagy pathways. Radioresistant changes in 6‐10B (B) and Tu686 (C) cells overexpressing Wnt3a or control cDNA were irradiated with the indicated doses of irradiation and survival fraction curves were obtained. Forced expression of Wnt3a in 6‐10B (D) and Tu686 (E) cells led to corresponding changes in γH2AX foci staining. *P < 0.05; **P < 0.01; ***P < 0.001

rhWnt3a protein was then used to activate the Wnt signalling pathway in both Tu686 and 6‐10B cells, which was confirmed by increased expression of β‐catenin, c‐Myc and Survivin (Figure S2A). Meantime, Wnt activation increased the protein expression of Beclin1 and LC3B (Figure S2A), indicating that Wnt3a‐mediated Wnt activation induced autophagy in SCCHN cells. Additionally, activation of the Wnt signalling pathway correspondingly led to enhanced resistance of 6‐10B and Tu686 cells to irradiation (Figure S2B and C). Consistent with the effects of exogenous rhWnt3a treatment, forced endogenous expression of Wnt3a in Tu686 and 6‐10B cells activated the Wnt signalling pathway (Figure 2A), up‐regulating Beclin1 (Figure 2A). Wnt3a overexpression led to a clear decrease in the formation of γH2AX foci (Figure 2D and 2), indicating an enhanced capacity to repair DNA double‐strand breaks, which is in accordance with the enhanced radioresistance in Tu686 and 6‐10B cells (Figure 2B and 2). Taken together, these data indicate that Wnt3a induces autophagy and enhances SCCHN radioresistance in vitro.

### 3.3 Autophagy confers radioresistance in SCCHN

**Figure 3:**
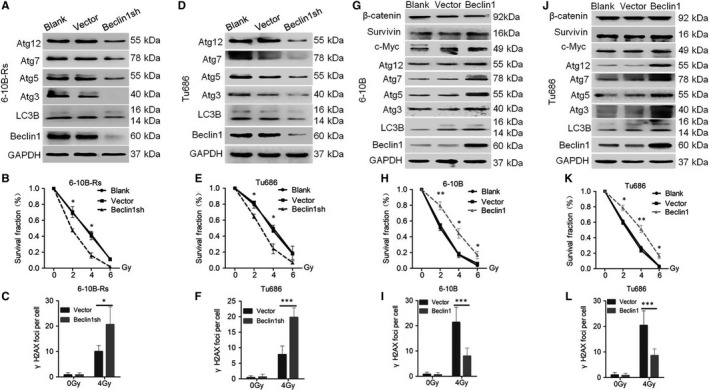
Autophagy confers radioresistance in SCCHN in vitro. (A‐F) SCCHN cells were infected with Beclin1 to inhibit its expression and then Western blotting assays were applied to quantify the levels of proteins associated with the Wnt and autophagy signalling pathways in 6‐10B‐Rs (A) and Tu686 (D) cells. Clonogenic assays were used to determine the survival fraction of 6‐10B‐Rs (B) and Tu686 (E) cells. Immunofluorescence staining was conducted to quantify γH2AX foci in 6‐10B‐Rs (C) and Tu686 (F) cells. SCCHN 6‐10B (G‐I) and Tu686 (J‐L) cells were forced to express Beclin1 and then proteins associated with the Wnt and autophagy signalling pathways were examined by Western blotting assays (G, J). Clonogenic assays were conducted to determine the survival fraction of 6‐10B (H) and Tu686 (K) cells. Immunofluorescence staining was used to quantify γH2AX foci in 6‐10B‐Rs (I) and Tu686 (L) cells. *P < 0.05; **P < 0.01; ***P < 0.001

Autophagy functions in a dual manner in cancer malignant progression.^8^, ^9^ To confirm the precise protective role of autophagy in SCCHN radioresistance, the expression of Beclin1 was inhibited in 6‐10B‐Rs and Tu686 cells (Figure 3A and 3). Beclin1 knockdown successfully inhibited the downstream autophagic signalling pathway, reflected by decreased expression of Atg3, Atg5, Atg7, Atg12 and LC3B (Figure 3A and 3), but showed no effect on the expression of β‐catenin, c‐Myc and Survivin (Figure S3A), indicating that Beclin1‐mediated autophagy did not regulate the Wnt signalling pathway. More importantly, Beclin1 knockdown re‐sensitized Tu686 and 6‐10B‐Rs cells to irradiation (Figure 3B and 3; Figure S3B), which was accompanied by a significantly increased number of γH2AX (Figure 3C and 3; Figure S3C). In contrast, without the influence on the Wnt signalling pathway, Beclin1 overexpression in Tu686 and 6‐10B cells activated the autophagic proteins Atg3, Atg5, Atg7, Atg12 and LC3B, inhibited the formation of γH2AX and led to SCCHN radioresistance (Figure 3G‐L; Figure S3B and C). Consistent with the data obtained from Beclin1 modulation, the autophagy inhibitor 3MA also restored the radiosensitivity of 6‐10B‐Rs (Figure S4A and 4B) and Tu686 (Figure S4D and E) in vitro, while 3MA did not regulate the expression of Beclin1 or proteins associated with the Wnt signalling pathway (Figure S4C and F).

**Figure 4:**
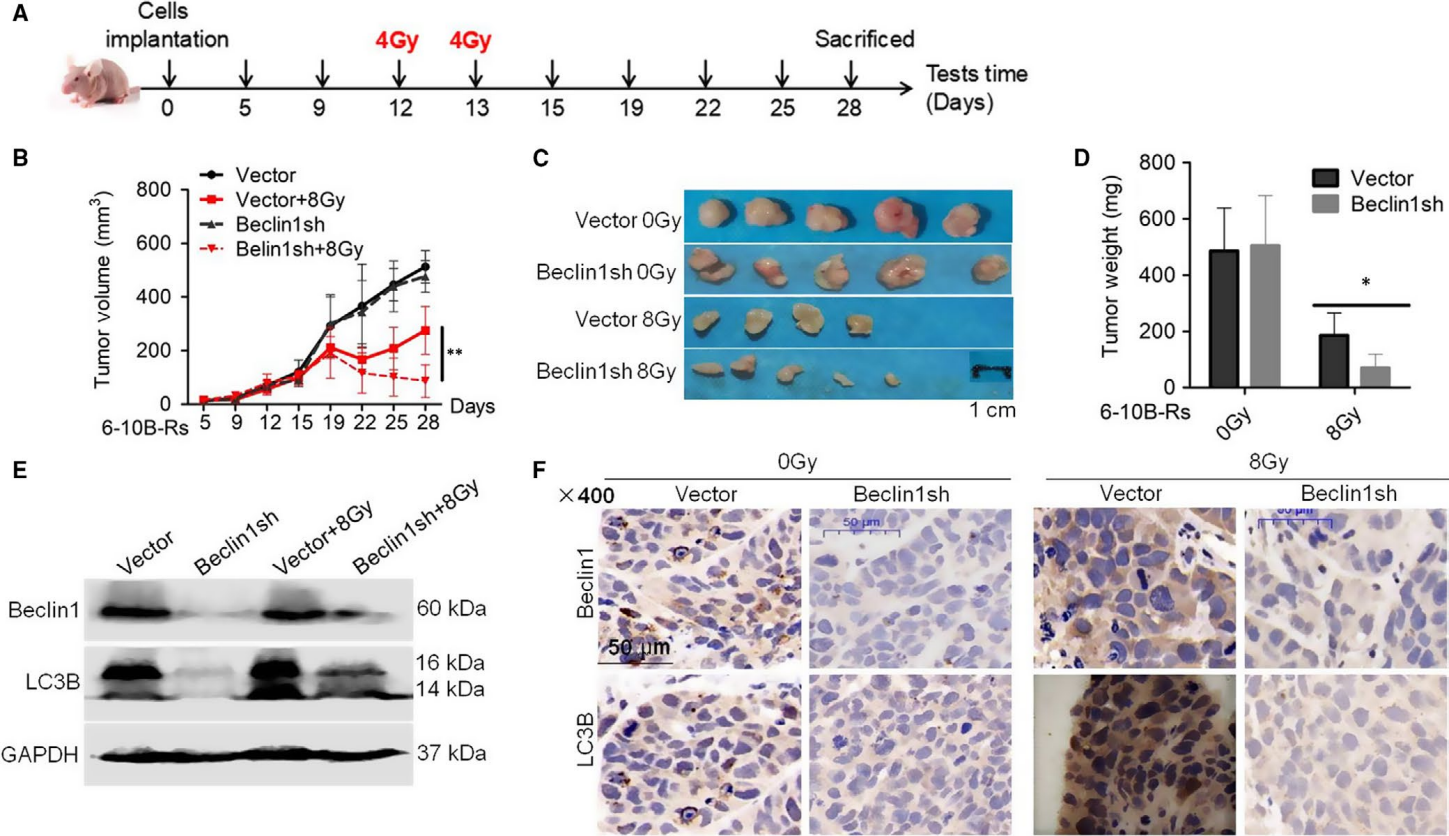
Autophagy confers radioresistance in SCCHN in vivo. (A) Schematic flow of the experimental design. Nude mice were subcutaneously injected with 6‐10B‐Rs cells expressing Beclin1 shRNA or not and then subjected to two consecutive 4 Gy irradiation treatments when the tumour volume reached 100 mm^3^. Mice were killed at day 28 after tumour injection. (B) Tumour volumes were measured with callipers every 3‐4 days to generate a curve of the growth pattern. (C) The final gross tumours were captured. (D) Average tumour weight in each group was compared. (E) Autophagic proteins Beclin1 and LC3B in each group were examined by Western blotting assays. (F) Immunohistochemistry staining was used to evaluate the expression of Beclin1 and LC3B. *P < 0.05; **P < 0.01; ***P < 0.001

To further confirm the above results in vivo, 6‐10B‐Rs cells infected with control and Beclin1 shRNA were subcutaneously injected into the flanks of nude mice. Mice were consecutively subjected to 4 Gy irradiation exposure twice and killed at 28 days after cancer cell injection (Figure 4A). Without radiotherapy, Beclin1 knockdown had no effect on the proliferation of 6‐10B‐Rs cells in vivo (Figure 4B‐D). However, Beclin1 knockdown sensitized 6‐10B‐Rs cells to a total of 8 Gy radiotherapy (Figure 4B‐D). Western blotting assays and immunohistochemistry (IHC) staining showed that Beclin1 and LC3B were efficiently inhibited in xenograft tumours (Figure 4E and 4). Taken together, our data indicate that inhibition of Beclin1‐mediated autophagy improves the therapeutic efficiency of radiotherapy in SCCHN.

### 3.4 Wnt3a enhances radioresistance via autophagy

**Figure 5:**
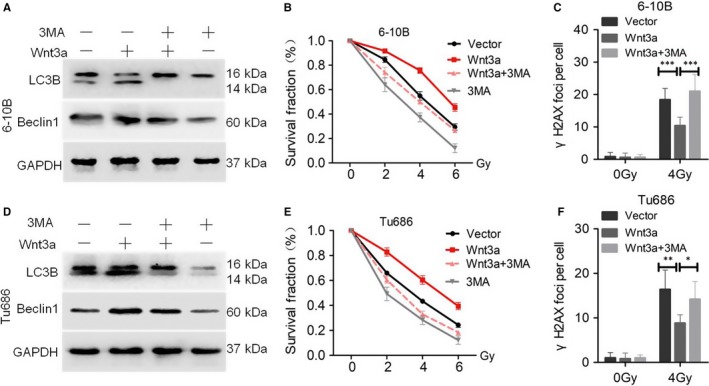
Wnt3a enhances radioresistance via autophagy in vitro. SCCHN 6‐10B (A‐C) and Tu686 (D‐F) cells were infected with lentivirus‐mediated Wnt3a or control cDNA and then exposed to 3MA treatment. (A, D) Western blotting assays were used to evaluate the expression LC3B and Beclin1. (B, E) Survival curves of cells in each group were determined by clonogenic assays. (C, F) γH2AX foci were quantified by immunofluorescence staining. *P < 0.05; **P < 0.01; ***P < 0.001

The above data clearly reveal that both the Wnt and autophagic signalling pathways contribute to SCCHN radioresistance. Therefore, we examined whether the Wnt pathway promotes radioresistance by modulating autophagy. To evaluate this, 6‐10B and Tu686 cells transfected with Wnt3a cDNA were treated with 3MA or left untreated and then the changes in radioresistance were evaluated. As shown in Figure 5A and D, 3MA blocked Wnt3a‐induced up‐regulation of LC3B. More importantly, 3MA also reversed the decreased γH2AX and radioresistance caused by rhWnt3a (Figure 5B, C, E, and F; Figure S5A‐D), suggesting that protective autophagy participates in the radioresistance enhanced by the canonical Wnt signalling pathway. However, 3MA is an inhibitor that acts on the late phage of the autophagic signalling pathway, while Beclin1 functions in the early stage of autophagy. We found that 3MA did not influence the level of Beclin1 protein (Figure 5A and D).

**Figure 6:**
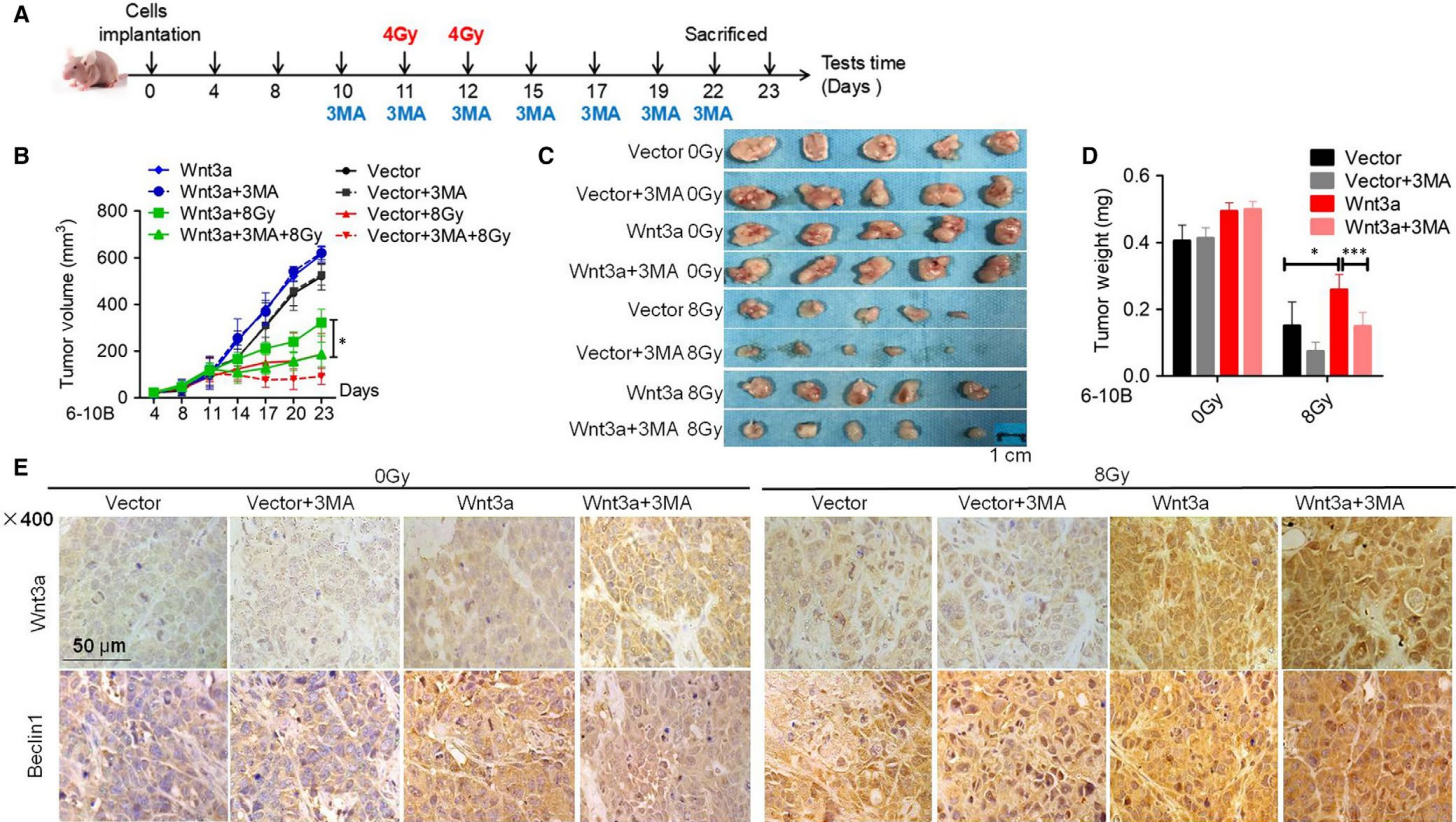
Wnt3a enhances radioresistance via autophagy in vivo. (A) Schematic flow of the experimental design. Nude mice were subcutaneously injected with 6‐10B cells overexpressing Wnt3a and then subjected to two consecutive 4 Gy irradiation treatments and 3MA treatment when the tumour volume reached 100 mm^3^. Mice were finally killed at day 23 after tumour injection. (B) Tumour volumes were measured with callipers every 3‐4 days to monitor the growth pattern of mice. (C) The final gross tumours were captured. (D) Average tumour weight in each group was compared. (E) Immunohistochemistry staining was used to examine the expression of Wnt3a and Beclin1. *P < 0.05; **P < 0.01; ***P < 0.001

Next, we repeated the experiments in vivo. 6‐10B cells transfected with control or Wnt3a cDNA were used to establish xenograft tumours. When the tumour volume reached 100 mm^3^, the mice were randomly divided into eight groups (5 mice in each group) and subjected to 8‐Gy radiotherapy and treatment with the autophagy inhibitor 3‐MA (Figure 6A). We found that Wnt3a improved the survival advantage radiotherapy‐treated mice compared to in mice without irradiation exposure (Figure 6B‐D). Similar to the in vitro results, 3‐MA effectively impeded the up‐regulation of Beclin1 (Figure 6E) and significantly restored radiosensitivity in Wnt3a‐overexpressing 6‐10B cells (Figure 6B‐D). Collectively, these data reveal that Wnt3a‐mediated activation of the canonical Wnt signalling pathway promotes SCCHN radioresistance via protective autophagy.

### 3.5 Clinical relevance of Wnt3a and Beclin1 in SCCHN patients

**Figure 7:**
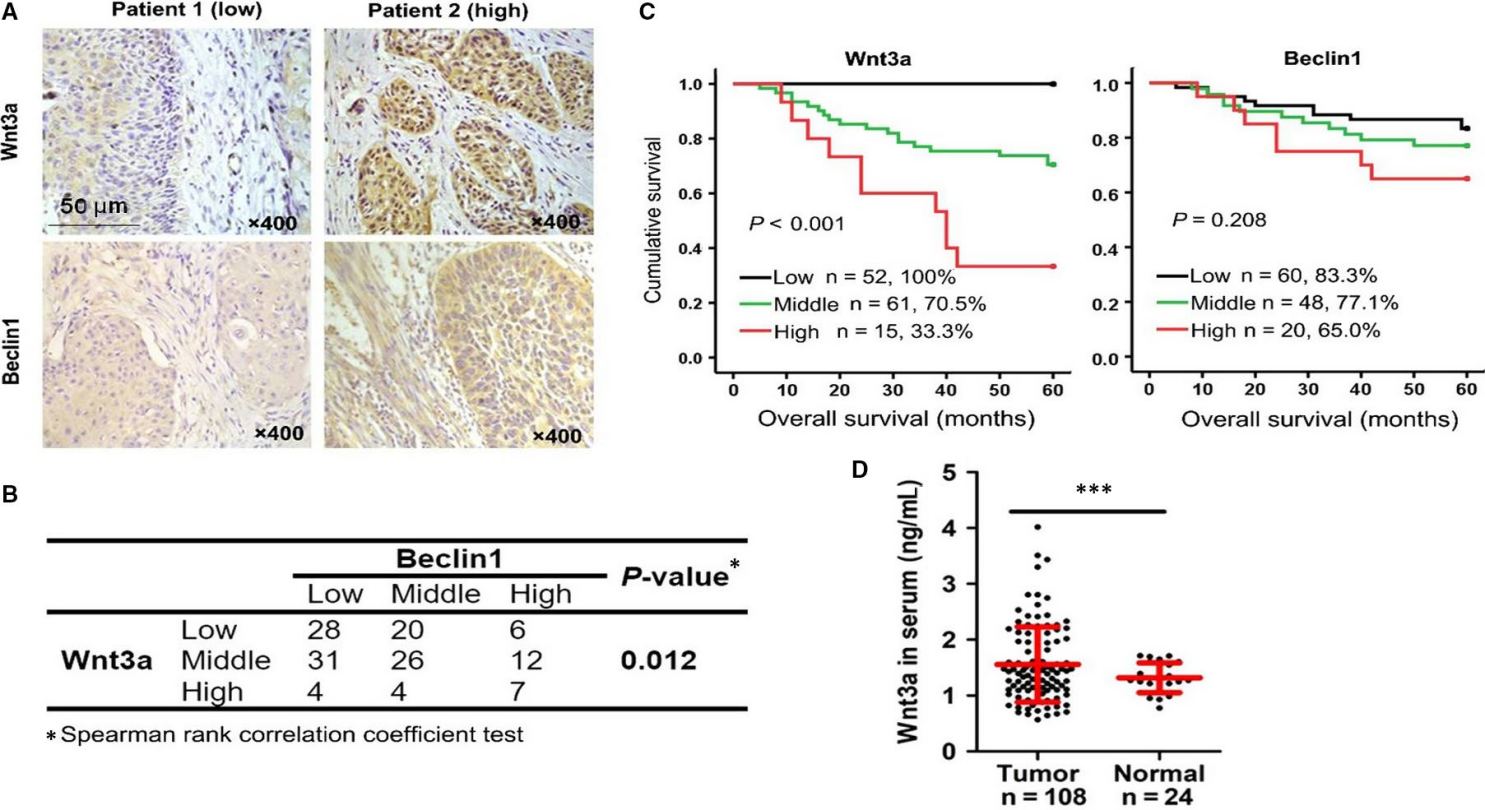
Clinical relevance of Wnt3a and Beclin1 in SCCHN patients. (A) Expression of Wnt3a and Beclin1 was examined by IHC in 138 samples from patients with SCCHN. Representative IHC images of Wnt3a and Beclin1 are shown, in which patient 1 displayed low expression of Wnt3a and Beclin1, while patient 2 had high expression. (B) The Spearman rank correlation coefficient validated the positive correlation between Wnt3a and Beclin1. (C) Kaplan‐Meier survival analysis of overall survival in all patients according to the expression of Wnt3a or Beclin1 in 128 patients with intact prognostic information. The log‐rank test was used to calculate the *P*‐value. (D) Serum Wnt3a was quantified by ELISA in 108 samples from patients with SCCHN and 24 healthy donors. ***P < 0.001

Finally, IHC was conducted to evaluate the expression of Wnt3a and then correlated with clinicopathological parameters in 138 patients with SCCHN. As shown in Figure 7A and B, Wnt3a and Beclin1 expression levels were positively correlated in SCCHN tissues. Wnt3a expression was positively correlated with lymph node metastasis and clinical stages and negatively associated with poor prognosis (Figure 7C; Table 1), indicating their potential value in the surveillance of cancer progression and prognosis. Wnt3a, a secretory protein, can be detected in patient serum. Therefore, serum Wnt3a was also quantified in patients with SCCHN. Although we found that serum Wnt3a levels in patients with SCCHN were much higher than those in healthy donors (Figure 7D) and its expression level was not correlated with multiple clinical parameters (Table S3). Beclin1 was mildly increased in patients with SCCHN at late clinical stages, however, we did not determine the clinical significance of Beclin1 in patients with SCCHN (Figure 7A and B; Table S2). Univariate Cox regression analyses determined that T classifications, clinical stages and Wnt3a protein levels were significantly associated with overall survival status in patients with SCCHN. However, only Wnt3a expression was an independent prognostic factor via multivariate Cox regression analyses (Table 2). Thus, our clinical data suggest that Wnt3a not only impacts SCCHN radioresistance, but also has potential value for the surveillance of SCCHN progression and prognosis.

**Table 2:** The overall survival Cox regression analysis

	Relative risk (95% CI）	*P*‐Value^a^
Univariate		
Sex	21.805 (0.044‐34.441 × 104)	0.412
Age	1.295 (0.612‐2.738)	0.499
Primary tumour sites	1.576 (0.745‐3.332)	0.234
T classifications	2.600 (1.105‐6.118)	**0.029**
Lymph node metastasis	2.018 (0.960‐4.241)	0.064
Clinical stages	3.939 (1.366‐11.357)	**0.011**
Histological grades	0.433 (0.282‐1.718)	0.433
Wnt3a expression	5.060 (2.832‐9.042)	**0.000**
Beclin1 expression	1.417 (0.875‐2.297)	0.157
Multivariate		
T classification	1.157 (0.377‐3.554)	0.799
Clinical stages	1.422 (0.738‐2.738)	0.293
Wnt3a expression	4.432 (2.421‐8.116)	**0.000**

a *P*‐values in bold were statistically significant. Abbreviation: 95% CI: 95% confidence interval.

## 4 DISCUSSION

In the current study, SCCHN cells with high activity of the Wnt signalling pathway are more resistant to radiotherapy and inactivation of the Wnt signalling pathway radiosensitizes SCCHN cells both in vitro and in vivo. Our data reveal the role of Wnt‐mediated autophagy in radioresistance and indicate that the Wnt pathway confers radioresistance to SCCHN cells through protective autophagy, suggesting that dual targeting of both the Wnt and autophagy pathways is useful for improving the effectiveness of radiotherapy.

Irradiation exposure enriches cancer stem cell‐like progenitor cells when the Wnt signalling pathway is highly activated.^24^ The Wnt pathway can induce both epithelial‐mesenchymal transition and cancer stem cell‐like properties, playing a key role in cancer malignant proliferation and therapy resistance,^25^, ^26^ which also occurs in patients with SCCHN.^27^ Thus, the Wnt pathway may contribute to cancer radioresistance by inducing the epithelial‐mesenchymal transition and maintaining cancer stem cell‐like properties, which requires future verification in SCCHN radioresistance. In contrast, autophagy inhibition has been shown to improve cancer radiosensitivity in preclinical investigations.^11^ However, whether the mutual interaction between the Wnt and autophagic signalling pathways and whether this interaction can cooperate in cancer radioresistance remains unknown.

The Wnt signalling pathway was recently reported to suppress the autophagic pathway in cancer cells.^28^, ^29^ In osteosarcoma cells, the Wnt/β‐catenin pathway inhibits autophagy and then rescues gemcitabine resistance in vitro.^29^ Similarly, β‐catenin inhibition using siRNA blocks the Wnt signalling pathway and represses stress‐induced autophagy in colorectal carcinoma.^28^ However, Wnt3a induces autophagy via the GSK‐3β/AMPK pathway which bypasses the canonical Wnt signalling pathway in neuronal metabolism, suggesting a canonical Wnt signalling‐independent mechanism in Wnt3a‐mediated autophagy.^30^ In a radioresistant environment, Wnt3a increased the expression of Beclin1, which in turn promoted protective autophagy and finally facilitated the occurrence of SCCHN radioresistance. Our data clearly revealed that Wnt3a stimulates the Wnt/β‐catenin pathway and functions as a positive regulator of autophagy to enhance SCCHN radioresistance, which is consistent with the results observed in neurons. Collectively, our data combined with those of previous studies indicate that the Wnt signalling pathway induces or represses autophagy in a manner that depends on the cell type and its survival environment.

The above opposite results may also be explained by the observation that other specific potential signalling pathways except for the canonical Wnt/β‐catenin pathway are activated in distinct cell environments. For example, Wnt3a accelerates the autophagic level in neurons by modulating the GSK‐3β‐AMPK axis, but not the Wnt/β‐catenin pathway,^30^ indicating that a β‐catenin‐dependent or independent signal can generate cancer cells with a radioresistant phenotype. In this study, we only confirmed that Wnt3a activates the Wnt/β‐catenin pathway, which does not exclude the possibility that other latent signalling pathways are modulated following exposure to Wnt3a stimulation. Thus, these potentially activated pathways may enhance or restrain the effect of Wnt/β‐catenin. Additionally, the Wnt/β‐catenin pathway participates in cancer malignant behaviours by interacting with other signalling pathways including Notch1/3,^31^, ^32^ Hippo,^33^ nuclear factor‐κB,^4^ and extracellular signal‐regulated kinase 1/2,^34^ all of which were reported to have dual effects on autophagy. Finally, even the basic expression level of β‐catenin is associated with a favourable or unfavourable prognosis in several solid cancers,^35^ suggesting that canonical β‐catenin can act as an oncogene or cancer suppressor depending on the cancer type. Overall, these complicated downstream and/or mutual interaction pathways of Wnt signalling may lead to distinct roles in autophagy.

The Wnt ligands, of which there are more than 19 closely related but distinct secreted cysteine‐rich glycoproteins, have been characterized according to their roles in early development and tumourigenesis.^4^ Wnt3a, a canonical Wnt ligand, is elevated in hepatocellular carcinoma,^31^, ^36^, ^37^ prostate cancer,^38^ oesophageal squamous cell carcinoma,^39^ lung adenocarcinoma^25^ and colon or colorectal carcinoma.^26^, ^40^ Moreover, increased Wnt3a protein levels are tightly associated with multiple aggressive cancer phenotypes, such as metastasis, advanced clinical stages and worse patient survival status, revealing that Wnt3a is a valuable prognostic biomarker in human malignancy. Our data reveal that high expression of Wnt3a is significantly correlated with lymph node metastasis and advanced clinical stages, which is consistent with the results of previous studies in other human malignancies including hepatocellular carcinoma and lung adenocarcinoma.^25^, ^37^, ^39^

Serum Wnt3a level is a useful diagnostic biomarker for hepatocellular carcinoma, particularly in alpha fetoprotein‐negative hepatocellular carcinoma.^36^ Although serum Wnt3a was dramatically elevated in patients with SCCHN, no significant difference was observed between the levels of serum Wnt3a and clinical parameters of patients with SCCHN. It is possible that Wnt3a is secreted not only from cancer cells, but also from inflammatory cells in the cancer environment.^4^, ^41^ The serum Wnt3a level reflects its secretion from diverse cells. Additionally, hepatocellular carcinoma is rich in blood vessel‐ and tumour cell‐derived Wnt3a, which easily enters the circulating blood system. This is not the case in SCCHN, which is relatively absent of tumour angiogenesis.^42^

Clinically, Beclin1 has been reported to be positively or negatively correlated with a poor or better survival status in distinct solid malignancies.^43^, ^44^, ^45^ In this study, Beclin1 was found to act as an oncogene to promote radioresistance in patients with SCCHN. However, although expression of Beclin1 was mildly increased in patients with SCCHN at advanced clinical stages, no significant clinical associations were observed in our patient cohort.

Probability prediction of successful radiotherapy is very important for personalized treatment of patients with SCCHN. The current risk stratification system for patients with SCCHN is mainly based on tumour‐node‐metastasis stage, which is important for making clinical treatment strategy decisions, but this system must be improved. Reliable indicators of the response of patients with SCCHN to radiotherapy are urgently needed. Retrospective clinical investigations of numerous cancers demonstrated that potential biomarkers in SCCHN samples are important tools for predicting radiotherapy outcome and appropriate treatment selection.^46^ Particularly, combined with functional and molecular investigations of these biomarkers, these studies provide a foundation for the discovery of molecular targets for managing SCCHN radioresistance. Our data clearly indicate that Wnt3a is a useful biomarker for the surveillance of SCCHN progression and a potential target for resensitizing SCCHN cells to irradiation. Therefore, evaluating Wnt3a protein may provide valuable information for predicting the prognosis of patients with SCCHN. Additionally, such information will improve strategies for irradiation sensitization of patients with SCCHN.

In summary, our data indicate that Wnt3a, as a canonical ligand for the Wnt signalling pathway, binds to its receptor on the membrane of SCCHN cells, activates the canonical Wnt signalling pathway, accelerates the nuclear translocation of β‐catenin and then enhances the expression of Beclin1. Thus, increased Beclin1 promotes autophagy, which prevents DNA damage and the formation of γH2AX foci following exposure to irradiation, finally inducing radioresistance. This study not only revealed the signalling interaction by which Wnt3a‐mediated activation of the canonical Wnt signalling pathway induces protective autophagy and contributes to SCCHN radioresistance, but also provides a clinical opportunity involving a combinational target of the Wnt signalling pathway and autophagy for treating patients with SCCHN with radioresistance. Further studies are needed to determine whether these results can be extended to the management of other solid human cancers.

## CONFLICT OF INTEREST

The authors confirm that there are no conflict of interests.

## AUTHORS’ CONTRIBUTIONS

Yong Liu, Yuanzheng Qiu, Qiancheng Jing and Guo Li were responsible for the concept and experimental design. Qiancheng Jing, Guo Li, Xiyu Chen, Chao Liu, Shanhong Lu, Hua Zheng, Huiling Ma, Yuexiang Qin, Diekuo Zhang, Shuiting Zhang and Shuling Ren performed the experiments, data analysis and statistical analysis. Donghai Huang, Pingqing Tan, Jie Chen and Yuanzheng Qiu provided technical and material support. Yong Liu and Qiancheng Jing were involved in drafting and revision of the manuscript. Yong Liu and Yuanzheng Qiu supervised this study. All authors discussed the results and commented on the manuscript.
