# Supplementary figures and images for: Wnt3a promotes radioresistance via autophagy in squamous cell carcinoma of the head and neck
Source: J Cell Mol Med. 2019 May 21;23(7):4711–22. doi: 10.1111/jcmm.14394 (PMC6584592; doi:10.1111/jcmm.14394)

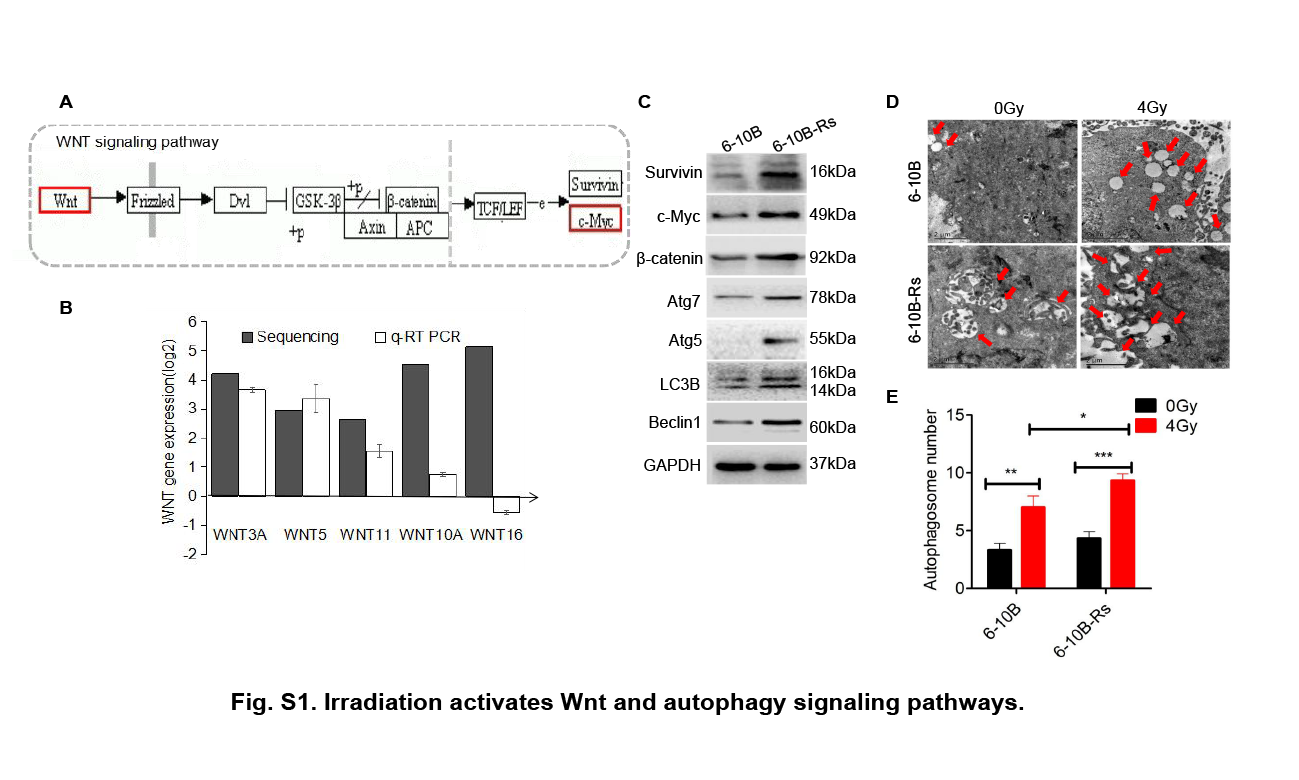

Supplement: Supplementary file 1 [file JCMM-23-4711-s001.tif]

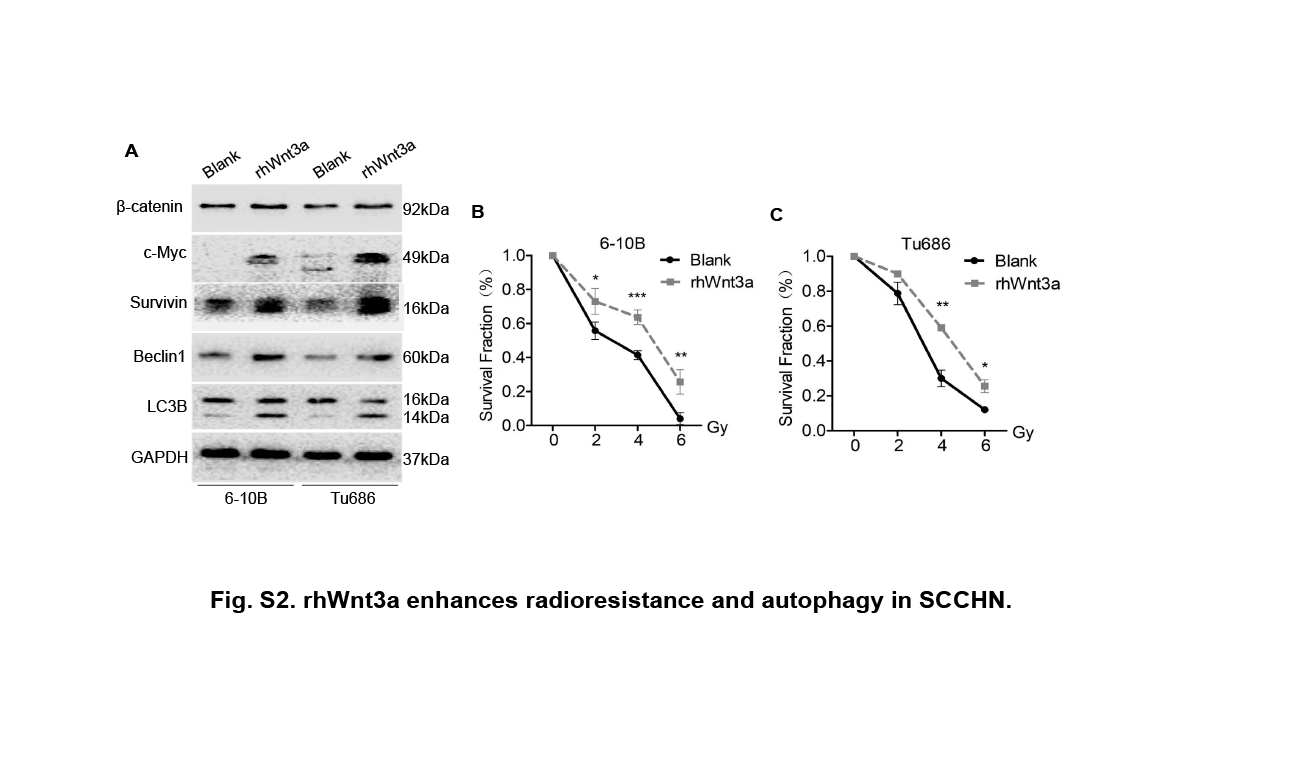

Supplement: Supplementary file 2 [file JCMM-23-4711-s002.tif]

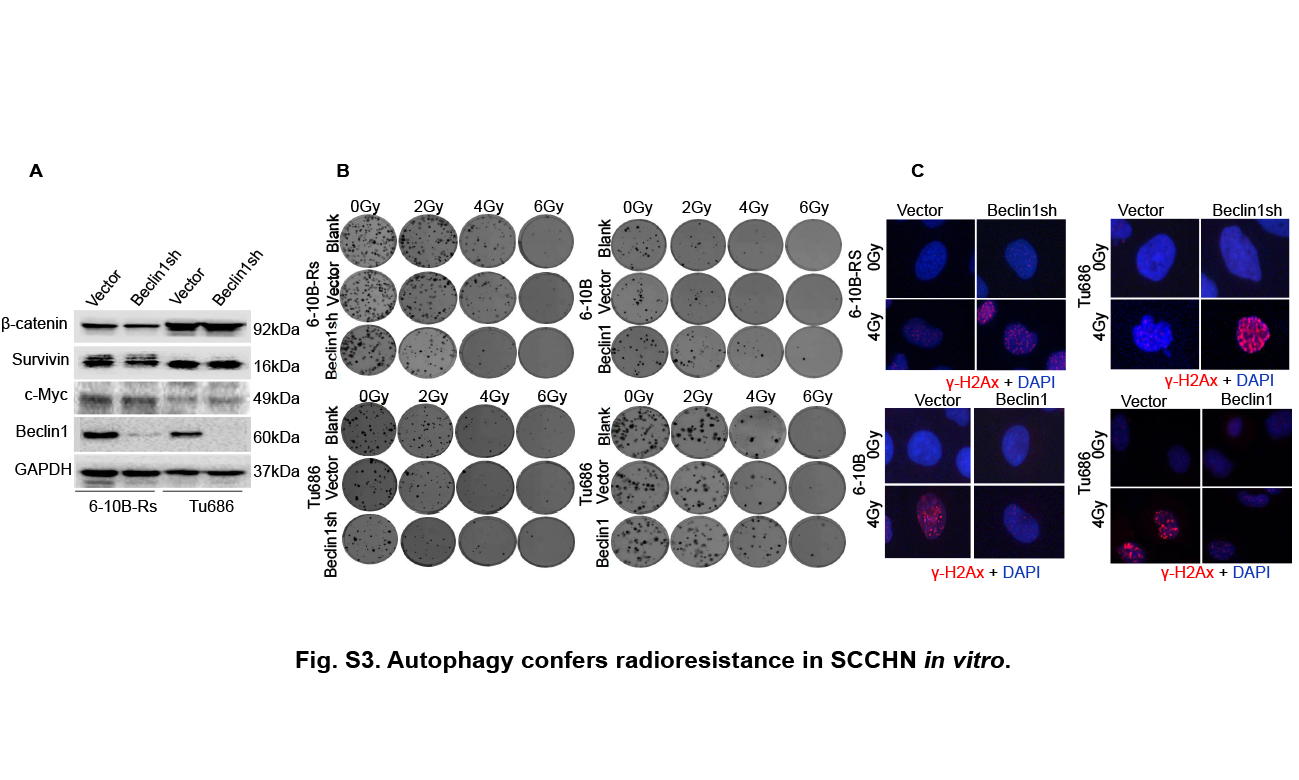

Supplement: Supplementary file 3 [file JCMM-23-4711-s003.tif]

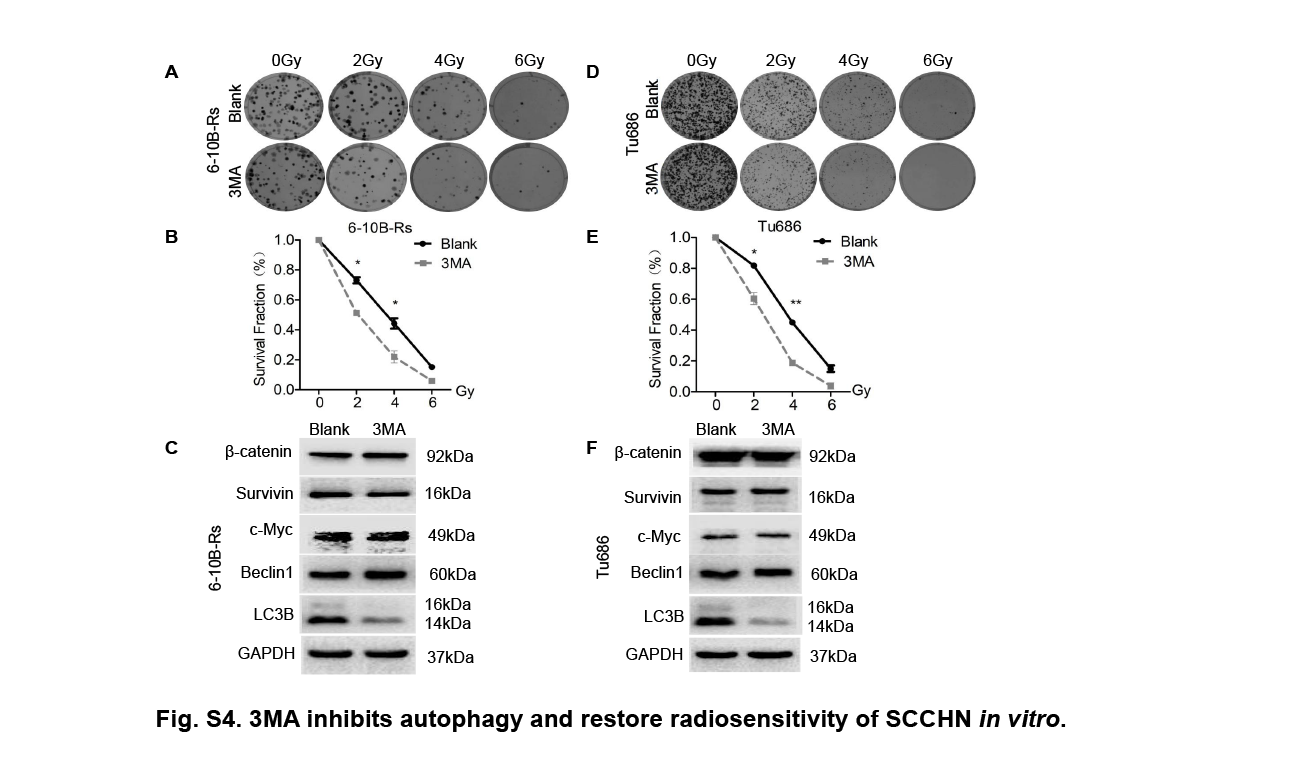

Supplement: Supplementary file 4 [file JCMM-23-4711-s004.tif]

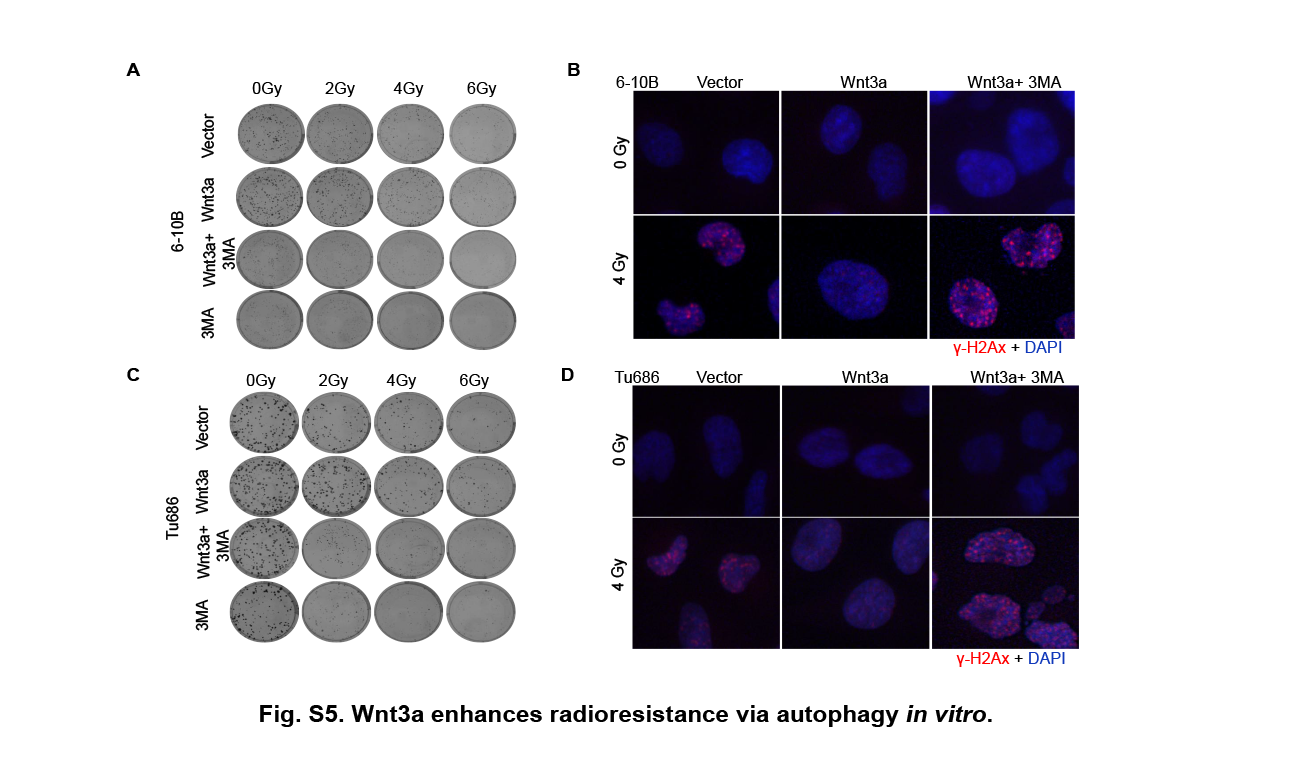

Supplement: Supplementary file 5 [file JCMM-23-4711-s005.tif]
