# Supplementary material for: Wnt3a promotes radioresistance via autophagy in squamous cell carcinoma of the head and neck
Source: J Cell Mol Med. 2019 May 21;23(7):4711–22. doi: 10.1111/jcmm.14394 (PMC6584592; doi:10.1111/jcmm.14394)
Supplement: Supplementary file 6 [file JCMM-23-4711-s006.docx]

**Table S1 The antibody information.**

| Antibody | Company | NO. | Working concentration | |
| --- | --- | --- | --- | --- |
| \| Survivin \| \| --- \| \| c-Myc \| \| Wnt3a \| \| β-catenin \| \| LC3B \| \| LC3B \| \| Beclin1 \| \| Beclin1 \| \| γH2AX \| \| Atg3 \| \| Atg5 \| \| Atg7 \| \| Atg12 \| \| GAPDH \| \| Goat anti-  rabbit IgG \| \| Donkey  Anti-Rabbit IgG \| | \| Affinity \| \| --- \| \| Affinity \| \| abcam \| \| Cell Signaling Technology,USA \| \| Beyotime Inc, China \| \| Cell Signaling Technology,USA \| \| abcam \| \| Cell Signaling Technology,USA \| \| [Novus Biologicals, Inc,USA](http://novusbio.lookchem.com/) \| \| Cell Signaling Technology,USA \| \| Cell Signaling Technology,USA \| \| Cell Signaling Technology,USA \| \| Cell Signaling Technology,USA \| \| Cell Signaling Technology,USA \| \| Beyotime Inc, China \| \| Beyotime Inc, China \| | \| AF6017 \| \| --- \| \| AF0358 \| \| ab81614 \| \| 8480S \| \| AL221 \| \| 3868S \| \| ab114071 \| \| D40C5 \| \| NB100-384 \| \| 3415S \| \| 12994S \| \| D12B11 \| \| D88H11 \| \| 5174 \| \| A0208 \| \| P0179 \| | \| 1:1000 (WB) \| \| --- \| \| 1:1000 (WB) \| \| 1:1000 (WB),1:400 (IHC) \| \| 1:1000 (WB),1:100 (IF) \| \| 1:400 (IF) \| \| 1:1000 (WB) \| \| 1:200 (IHC) \| \| 1:1000 (WB) \| \| 1:4000 (IF) \| \| 1:1000 (WB) \| \| 1:1000 (WB) \| \| 1:1000 (WB) \| \| 1:1000 (WB) \| \| 1:1000 (WB) \| \| 1:2000 (WB),1:100 (IHC) \| \| 1:500 (IF) \| | |
|  |  |  |  |  |

**Table S2 Correlation of Beclin1 expression with clinicopathological features in 138 patients with SCCHN.**

| Parameters | Low | Middle | High | *χ2* value | *P*-value^1^ |
| --- | --- | --- | --- | --- | --- |
| Sex |  |  |  |  |  |
| Female | 3 | 3 | 0 | 1.491 | 0.475 |
| Male | 60 | 47 | 25 |  |  |
| Age |  |  |  |  |  |
| <58 | 28 | 27 | 13 | 1.109 | 0.574 |
| ≥58 | 35 | 23 | 12 |  |  |
| Primary tumor sites |  |  |  |  |  |
| Glottic | 45 | 30 | 17 | 1.663 | 0.435 |
| Others | 18 | 20 | 8 |  |  |
| T classifications |  |  |  |  |  |
| T1 + T2 | 29 | 20 | 12 | 0.590 | 0.745 |
| T3 + T4 | 34 | 30 | 13 |  |  |
| Lymph node metastasis |  |  |  |  |  |
| N0 | 46 | 34 | 15 | 1.439 | 0.487 |
| N+ | 17 | 16 | 10 |  |  |
| Histological grades |  |  |  |  |  |
| G1+G2 | 44 | 36 | 18 | 0.078 | 0.962 |
| G3+G4 | 19 | 14 | 7 |  |  |
| Clinical stages |  |  |  |  |  |
| Ⅰ | 3 | 4 | 2 | 2.652 | 0.851 |
| Ⅱ | 21 | 14 | 6 |  |  |
| Ⅲ | 26 | 22 | 9 |  |  |
| Ⅳ | 13 | 10 | 8 |  |  |

^1^ *P* < 0.05 was considered to be statistically significant.

**Table S3 Correlations between serum Wnt3a levels and clinicopathological parameters in 108 patients with SCCHN**

| Parameters | Low | High | *χ2* value | *P*-value^1^ |
| --- | --- | --- | --- | --- |
| Sex |  |  |  |  |
| Female | 1 | 5 | 0.794 | 0.373 |
| Male | 35 | 67 |  |  |
| Age |  |  |  |  |
| <58 | 18 | 30 | 0.675 | 0.411 |
| ≥58 | 18 | 42 |  |  |
| T classifications |  |  |  |  |
| T1 + T2 | 16 | 38 | 0.667 | 0.414 |
| T3 + T4 | 20 | 34 |  |  |
| Lymph node metastasis |  |  |  |  |
| N0 | 18 | 39 | 0.167 | 0.683 |
| N+ | 18 | 33 |  |  |
| Metastasis |  |  |  |  |
| M0 | 35 | 69 | 0.130 | 0.719 |
| M1 | 1 | 3 |  |  |
| Histological grade |  |  |  |  |
| G1/2 | 18 | 24 | 2.805 | 0.094 |
| G3/4 | 18 | 48 |  |  |
| Clinic stage |  |  |  |  |
| Ⅰ | 9 | 8 | 4.972 | 0.174 |
| Ⅱ | 5 | 20 |  |  |
| Ⅲ | 10 | 21 |  |  |
| Ⅳ | 12 | 23 |  |  |

^1^ *P* < 0.05 was considered to be statistically significant.
